# Recurrent Fevers in a Triathlete

**DOI:** 10.7759/cureus.12564

**Published:** 2021-01-07

**Authors:** Rupinder Mangat, Angelina Winbush, Ted Louie

**Affiliations:** 1 Division of Infectious Diseases, University of Rochester Medical Center, Rochester, USA; 2 Division of Infectious Diseases, University of Rochester School of Medicine and Dentistry, Rochester, USA

**Keywords:** anaplasma, anaplasmosis, recurrent fever

## Abstract

Human granulocytic anaplasmosis (HGA) is a tick-borne illness that typically causes a self-limited febrile illness. We describe herein a healthy triathlete who had recurrent fevers for six weeks as a result of HGA and discuss the impact of this patient’s HGA on his metabolic fitness during training over a three month time period. We also review the literature for other cases of Ehrlichia and Anaplasma with prolonged courses.

## Introduction

*Anaplasma phagocytophilum* is an obligate gram-negative, intracellular bacterium that causes human granulocytic anaplasmosis (HGA), formerly known as human granulocytic ehrlichiosis (HGE). This organism is transmitted by *Ixodes scapularis*, the tick that also transmits Lyme disease and Babesiosis. Cases are largely confined to the upper Midwest and Northeast in the United States, although cases have been reported worldwide. Tick-borne illnesses are on the rise, and therefore HGA is an important diagnosis to consider for patients with undifferentiated fevers and tick exposure in endemic areas [1].

Anaplasmosis typically presents as an acute illness with nonspecific symptoms such as fever, malaise, and headaches. While many patients resolve their symptoms spontaneously without treatment, some patients develop a severe infection, necessitating hospitalization [1]. Prolonged cases of anaplasmosis are rarely reported in the literature [2, 3]. Here, we present a case of anaplasmosis in a previously healthy male who experienced recurrent fevers for over six weeks before the diagnosis was made. 

## Case presentation

An active 50-year-old male with no significant past medical history was admitted to our hospital in early July of 2019 with recurrent fevers of uncertain etiology. The patient was in his usual state of good health until May 2019, when he started experiencing fevers and rigors associated with a “crushing” frontal headache, myalgias, and a transient rash on his torso while training for a triathlon. These symptoms were debilitating for the patient, leaving him unable to work or exercise. Blood work revealed leukopenia with Pelger-Huet cells and reactive lymphocytes, thrombocytopenia, and mild transaminitis (see Table 1). Further lab work drawn by his primary care physician during the episode demonstrated a mildly elevated C-reactive protein (CRP), antinuclear antibody (ANA) positive 1:80, and negative rheumatoid factor and Lyme titers. Although his symptoms and labs began to improve without any intervention, he had another episode of fever, headache, and myalgias for 2-3 days in mid-June and then again in late June into early July. Results of the additional labwork drawn in early and late June are also shown in Table 1. He was admitted to the hospital in early July for an additional workup of his symptoms.

**Table 1:** Laboratory results over time from our case study patient with anaplasmosis AST - aspartate aminotransferase; ALT - alanine aminotransferase; WBC - white blood cells

Lab tests	Late May 2019	Early June 2019	Late June 2019
AST (U/L)	178	27	29
ALT (U/L)	142	66	20
WBC (x10^3^/µL)	2.2	7.9	5.9
Platelets (x10^3^/µL)	72	179	232

Upon admission, the patient's blood pressure was 128/70, heart rate 85, respiratory rate 18, and temperature 37.5 degrees Celsius. Physical exam revealed a mildly ill patient, with normal heart, lung, abdominal, neurologic, and joint exam, and no rashes. Infectious diseases was consulted for assistance in evaluation and treatment.

The patient was born in Guyana and emigrated to the United States at the age of seven. He reported traveling to Lake George, NY on April 25 and discovering a tick embedded in his left elbow. About one week later, he went to Onondaga Park for the Saratoga Regatta, with additional potential tick exposure. He denied having any sick contacts, animal exposures, or unusual dietary habits. Family history was notable for an aunt with rheumatoid arthritis and a father with idiopathic pulmonary fibrosis.

Given a clinically high suspicion for tick-borne illness, the patient was empirically started on doxycycline 100 mg by mouth twice daily while awaiting additional testing. He began to experience an improvement in symptoms within 24 hours and thereafter remained afebrile. Labs revealed a highly positive Anaplasma phagocytophilum antibody test (immunoglobulin G [IgG] >1:1280 and immunoglobulin M [IgM] >1:16). Babesia, Ehrlichia, and cytomegalovirus (CMV) PCR were negative. Lyme and HIV antibodies, as well as blood cultures, were negative. 

The patient continued to feel fatigued for several weeks afterward. Interestingly, our patient uses TrainingPeaks - online tool athletes can use to track progress and reach their goals. At our request, the patient allowed us access to his training data. Per TrainingPeaks, the intensity of workouts gives a training stress score (TSS). "Fatigue" is based on a seven-day average of TSS, while "fitness" is based on a 42 day average of TSS. "Form" is calculated by "fitness" minus "fatigue". His fatigue, fitness, and form parameters each showed a bimodal distribution during his illness, with worsening form and fitness between mid-May and mid-June, and again from early July until August. Although feeling fatigued, he was able to train for a marathon in August, so gradually, by the end of August, his form and fitness had returned to his baseline. So in this particular patient, we have good data showing that untreated Anaplasmosis can cause significant, objective impairment in physical fitness over a three-month time period. 

## Discussion

Cases of Anaplasma phagocytophilum infections have increased substantially over the last several decades, with 348 recorded cases in 2000 and a peak of 5,762 cases recorded in 2017. The geographical range of cases is also growing, secondary to the expanding habitat of the Ixodes scapularis tick [4]. Importantly, the Ixodes scapularis tick is known to serve as a vector for multiple additional infectious diseases, including Lyme disease, Powassan virus, ehrlichiosis, babesiosis, and Borrelia miyamotoi infections. Lab abnormalities such as leukopenia (<4,500/mm^3^), thrombocytopenia (<150,000/mm^3^), and elevated aminotransferase levels are important indicators of the disease, all of which were present in our patient on initial lab testing. However, it is important to note that lab abnormalities commonly normalize by the second week, a phenomenon observed in our patient, as normal labs would not exclude the diagnosis [1]. While many cases of Anaplasmosis are subclinical in nature, between 33%-50% of symptomatic cases, require hospitalization and between 3-7% of this population will develop life-threatening complications [5]. Thus, Anaplasma is an important diagnosis to consider in a patient presenting with fever and tick exposure.

Diagnosis of Anaplasma is often made by a polymerase chain reaction, with sensitivity between 74-100% [6, 7]. Alternatively, antibodies can be measured by immunofluorescence. Positive IgM is often thought to be a result of acute illness, although this is not always reliable. A fourfold increase in IgG, measured by acute and convalescent titers, is considered diagnostic [8]. Hansmann et al. describe a strategy of combining acute and convalescent titers, or a single positive titer of greater than or equal to 1:256, as having a sensitivity of 58% and a specificity of 97% [7]. Our patient had borderline positive IgM and extremely high IgM late in his presentation. Our interpretation is that enough time had elapsed that his presumably elevated IgM had sufficient time to decrease, while the IgG titer had time to increase, although we did not have serial antibody measurements to confirm this. 

Although most cases of HGA are mild and self-limited even without antibiotic treatment, our patient had recurrent fevers for over six weeks until he received a course of treatment with doxycycline. Such prolonged fevers due to Anaplasma or Ehrlichia have rarely been described [1, 2].

In 1996, Dumler and Bakken published a report on HGE infections in patients living in Minnesota and Wisconsin. They obtained serum from 54 patients with undifferentiated fever in the summer and fall months and observed positive serological reactivity with an E. equi indirect fluorescent antibody test in 11% of the samples. PCR testing was performed for antibody-positive samples, which demonstrated the persistence of E. equi DNA in up to 21 days post-initial fever in the population. They also noted that persistent HGE infections had been observed in animals, likely as an evolutionary adaptation of the infectious agent to increase its likelihood of tick transmission between hosts [2].

Roland et al. showed that six out of 41 patients diagnosed with ehrlichiosis between 1989 and 1993 had a prolonged febrile illness lasting between 17 and 51 days. The protracted disease course in these patients was thought to be due to both - lack of consideration of the diagnosis and failure to seek medical attention [3].

Others have proposed that Ehrlichia and Anaplasma may cause chronic symptoms following treatment. A longitudinal study by Ramsey et al. revealed that symptoms of fevers, chills, sweats, and fatigue were more often reported in a set of patients treated for anaplasmosis compared to a control population for up to three years after the onset of illness. The authors believed that these symptoms could be postinfectious, given the lack of serological evidence for a persistent infection [9]. In contrast, Lantos and Wormser, in their 2014 review of the literature, did not find evidence to support relapsing or chronic Anaplasma infection [10]. We are not aware of any data that supports prolonged use of antibiotics for such postinfectious symptoms, nor do we advocate this approach. 

Herein, we present a rare case of anaplasmosis in a previously healthy patient but developed recurrent fevers over several weeks. Our patient did seek medical attention; however, the diagnosis was not considered early on in his disease course, and it was not until the infectious diseases team was consulted that anaplasmosis and other tick-borne illnesses were tested for and empiric therapy with doxycycline initiated. Based on our patient’s presentation, we believe anaplasmosis is an important differential to consider in patients with relapsing fever and a history of tick exposure especially given the increasing prevalence of Anaplasma in the United States as well as the severe morbidity and mortality associated with this disease.

## Conclusions

Our case clearly demonstrates the need to consider the diagnosis of anaplasmosis when evaluating a patient with recurrent fevers in the appropriate epidemiologic setting. The lack of specificity of signs and symptoms can make it difficult to diagnose early on in the disease course. Patients should be started on empiric therapy based on a presumptive diagnosis if suspicion for anaplasmosis is high as no rapid testing is available. Diagnosis can be confirmed with serology, PCR, immunohistochemistry, culture, or blood smear in which morulae can occasionally be observed.
